# Long and isolated graphene nanoribbons by on-surface polymerization on Au(111)

**DOI:** 10.1038/s42004-023-01073-3

**Published:** 2023-12-06

**Authors:** Umamahesh Thupakula, We-Hyo Soe, Christian Joachim, Erik Dujardin

**Affiliations:** 1 Centre d’Élaboration de Matériaux et d’Études Structurales (CEMES), Centre National de la Recherche Scientifique (CNRS), Université de Toulouse, 29 Rue J. Marvig, BP 94347, 31055 Toulouse Cedex, France; 2 International Center for Materials Nanoarchitectonics (WPI-MANA), National Institute for Materials Science (NIMS), 1-1 Namiki, Tsukuba, Ibaraki, 305-0044 Japan; 3 Laboratoire Interdisciplinaire Carnot de Bourgogne, CNRS UMR 6303, Université de Bourgogne Franche-Comté, 9 Av. A. Savary, 21078 Dijon, France; 4 Present Address: Center for Quantum Nanoscience, Institute for Basic Science (IBS), Ewha Womans University, Seoul, 03760 Korea

**Keywords:** Scanning probe microscopy, Synthesis and processing, Synthesis of graphene

## Abstract

Low electronic gap graphene nanoribbons (GNRs) are used for the fabrication of nanomaterial-based devices and, when isolated, for mono-molecular electronics experiences, for which a well-controlled length is crucial. Here, an on-surface chemistry protocol is monitored for producing long and well-isolated GNR molecular wires on an Au(111) surface. The two-step Ullmann coupling reaction is sequenced in temperature from 100 °C to 350 °C by steps of 50 °C, returning at room temperature between each step and remaining in ultrahigh vacuum conditions. After the first annealing step at 100 °C, the monomers self-organize into 2-monolayered nano-islands. Next, the Ullmann coupling reaction takes place in both 1st and 2nd layers of those nano-islands. The nano-island lateral size and shape are controlling the final GNR lengths. Respecting the above on-surface chemistry protocol, an optimal initial monomer coverage of ~1.5 monolayer produces isolated GNRs with a final length distribution reaching up to 50 nm and a low surface coverage of ~0.4 monolayer suitable for single molecule experiments.

## Abstract (web summary)

The on-surface synthesis of graphene nanoribbons with control over their length and final surface coverage is desirable for electronic applications. Here, the authors outline a protocol to produce long and isolated graphene nanoribbons on an Au(111) surface, achieving lengths of up coverage down to ~0.4 monolayer, of potential value for mono-molecular electronics. to 50 nm and a low surface coverage down to ~0.4 monolayer, of potential value for mono-molecular electronics.

## Introduction

On-surface synthesized graphene nanoribbons (GNRs) are low electronic gap conjugated organic oligomers with chemically tunable electronic properties^1–3^. They are used for fabricating devices based on molecular nanomaterials^4–6^. By transferring on-surface ultrahigh vacuum (UHV) grown GNRs onto an insulating surface, device characteristics in a field effect transistor configuration were recently recorded^7,8^. The on-surface synthesis of isolated GNRs with a well-controlled length is also important for mono-molecular electronics, for example in measuring the current−voltage characteristics of a single long and isolated molecular wire^9–11^. The standard on-surface synthesis of 7-carbon atom wide armchair type GNR (7-aGNR) is a two-step process with the initial formation of polyanthryl oligomers, by the activation of an Ullmann coupling reaction, followed by a cyclodehydrogenation along the polyanthryl backbone^12,13^. With this two-step approach and by using 10,10′-dibromo-9,9′-bianthryl (DBBA) monomers on Au(111) surface, the typical length of 7-aGNRs is around ~10 nm^14^. Few rare, 25-nm-long isolated 7-aGNRs, at the tail of the GNR length distribution have been observed^14,15^. GNR lengths can reach up to ~40–50 nm in a densely packed surface arrangement when the final GNR coverage exceeds one monolayer (ML)^15^. GNR lengths ranging up to 200 nm^16–21^ have also been obtained for specific monomers and always in dense surface packing conditions, that are impractical for single molecule experiments^22–24^.

In this paper, an on-surface chemistry protocol is given to produce long, yet well-isolated 7-aGNR molecular wires. For this purpose, an optimal starting monomer precursor coverage of 1.5 ML is first identified. Next, a step-by-step annealing procedure is carried out on the Au(111) surface, with a detailed atomic scale analysis of all the chemical intermediates encountered at each temperature step. To perform this controlled on-surface synthesis and its atomic scale analysis on the same sample, it is essential not to break the UHV experimental conditions between the different on-surface synthesis steps. Otherwise, exposing the surface of the sample to ambient conditions, between successive protocol steps, for ex-situ treatments will lead to the irreversible deterioration of the previous on-surface synthesis steps. This will further require re-preparation of the sample surface in UHV, which will destroy the results of the previous steps. Maintaining the UHV conditions all along the on-surface synthesis protocol beneficiates the high-resolution atomic-scale performances of our low temperature-UHV 4-scanning tunneling microscopy (LT-UHV 4-STM), which enables us to characterize, in parallel, millimeter-separated locations on the same Au(111) surface^25,26^. While some of the observed intermediates, like the monomer desorption or the formation of the polyanthryl oligomer nano-islands, have already been reported, the complete temperature-dependent sequence of a 7-aGNRs on-surface synthesis is presented here without breaking the vacuum or changing the sample. It reveals the complete mechanism of the on-surface chemical reaction.

## Results

### The on-surface synthesis protocol

**Fig. 1:**
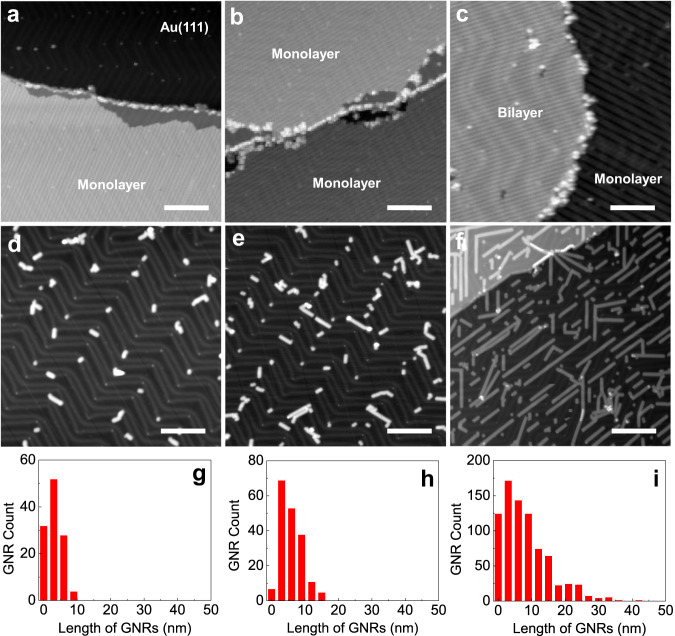
Effect of initial DBBA coverage on the final GNR lengths on the Au(111) surface. STM topographs showing the molecular self-assembled phases of DBBA at the initial coverages of (**a**) 0.5 ML, (**b**) 1.0 ML, and (**c**) 1.5 ML, on the Au(111) surface. **d**–**f** Corresponding STM topographs after the cyclodehydogenation step revealing the 7-aGNRs and (**g**–**i**) their length distribution histograms of the three samples shown in (**d**–**f**), respectively. STM set parameters are +2 V/10 pA for (**a**–**c**) and −2 V/10 pA for (**d**–**f**). Scale bars are 20 nm.

On an atomically flat metallic surface, the activation temperatures for the lateral diffusion of DBBA monomers and the formation of the chemical intermediates depend on the diffusion barrier of the monomers and their surface coverage. This coverage controls the kinetics of the on-surface chemical reaction^27^. When increasing the surface temperature, the polymerization of monomers is always in competition with the possible desorption of the monomers from the surface in UHV. Therefore, control annealing experiments were first conducted by using different samples with an initial DBBA surface coverage of 0.5 ML, 1.0 ML, 1.5 ML and > 2.0 ML on UHV-cleaned atomically flat Au(111) surfaces (Fig. 1a–c and Supplementary Fig. 1). These surface coverages were determined from the statistical analysis of large-scale STM images rather than from the nominal monomer evaporation rate out of the crucible.

After the polymerization (10 min, 200 °C) and cyclodehydrogenation (10 min, 400 °C) steps, the monomers were observed to be converted into 7-aGNRs for all coverages as presented in Fig. 1d–f and Supplementary Fig. 1. The length distributions extracted for all the coverages are given by the histograms presented in Fig. 1g–i and Supplementary Fig. 1. By varying the initial monomer coverage from 0.5 ML to 1.5 ML, we observed a fourfold increase of the maximum GNR lengths that remains constant when exceeding 2.0 MLs. A broadening of the GNR length distribution is also observed when increasing the initial monomer coverage. These DBBA coverage variation experiments point out a specific on-surface chemical kinetics that takes place when the DBBA surface coverage exceeds 1.0 ML. Therefore, the controlled step-by-step annealing experiments were performed for the selected sample of 1.5 ML initial DBBA coverage on the Au(111) surface. Note that other sublimation conditions do not significantly improve the final maximal lengths of the 7-aGNRs, while maintaining them in well-isolated configuration. (See Supplementary Fig. 12 for several other control experiments attempted to elongate the 7-aGNR length, while keeping them in an isolated surface configuration).

Stepwise annealing experiments at controlled temperature and under permanent UHV were performed on a given sample with 1.5 ML initial DBBA coverage. The annealing process was stopped at every 50 °C between 50 °C and 350 °C (step duration is 30 min) until well-defined 7-aGNRs were observed on the Au(111) surface. Following this on-surface reaction protocol, the recording of LT-UHV atomic scale STM imaging requires a systematic back-and-forth travel between our UHV preparation chamber and the LT-UHV 4-STM stage operated at ~4.5 K^26^. For each annealing step, the UHV travel is ~2 m long, each way, implying room and low temperature thermalizations for about 10 min (each way) between the LT-UHV 4-STM and the sample annealing stages. Therefore, after each annealing step in the UHV and during the room temperature thermalization of the sample, monomers certainly desorb from the Au(111) surface requiring also an intermediate cleaning of the UHV chambers. The annealing temperature intervals were determined by first increasing and then decreasing the temperature systematically, not to miss any essential chemical intermediates of the on-surface reaction. For our experimental setup and conditions (UHV traveling time and cleanliness of the transit chamber along the UHV path between the preparation and LT 4-STM stages), a 50 °C step was found to be optimum for the observation of the intermediate chemical reactions occurring on the same sample surface.

**Fig. 2:**
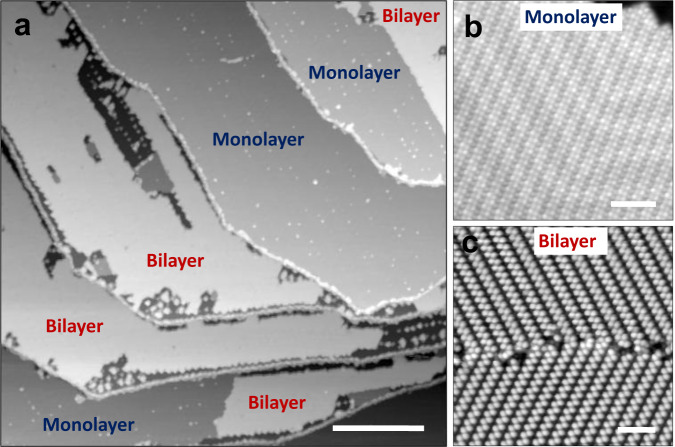
STM topographies of as-deposited 1.5 ML DBBA on Au(111). **a** Large-scale STM topograph showing the ML and BL regions of as-deposited 1.5 ML DBBA covered Au(111) surface. **b**, **c** High-resolution STM topographs showcasing the self-assembly patterns of DBBA in the ML and in the 2nd layer of BL regions, respectively. STM parameters are +2 V/10 pA. Scale bars are 100 nm for (**a**) and 5 nm for (**b**, **c**).

**Fig. 3:**
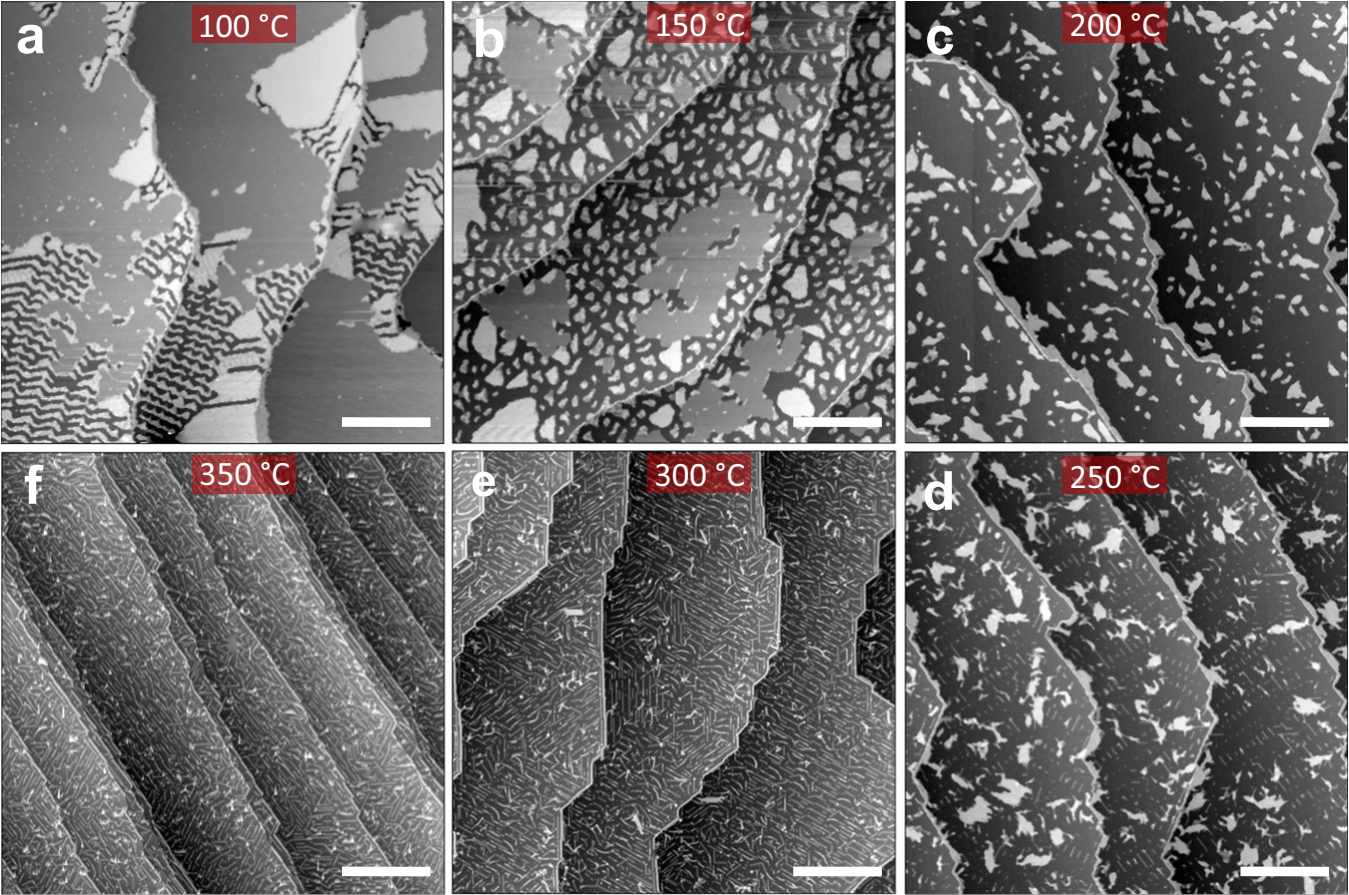
Modification of the nanoscale morphology by stepwise annealing of the same sample. Large-scale STM topographs revealing the annealing-induced surface modifications and structural phase changes in the 1.5 ML DBBA on the Au(111) surface at (**a**) 100 °C, (**b**) 150 °C, (**c**) 200 °C, (**d**) 250 °C, (**e**) 300 °C, and (**f**) 350 °C. STM set-parameters are +2 V/10 pA (**a**–**c**) and −2 V/10 pA (**d**–**f**). Scale bars are 100 nm.

**Fig. 4:**
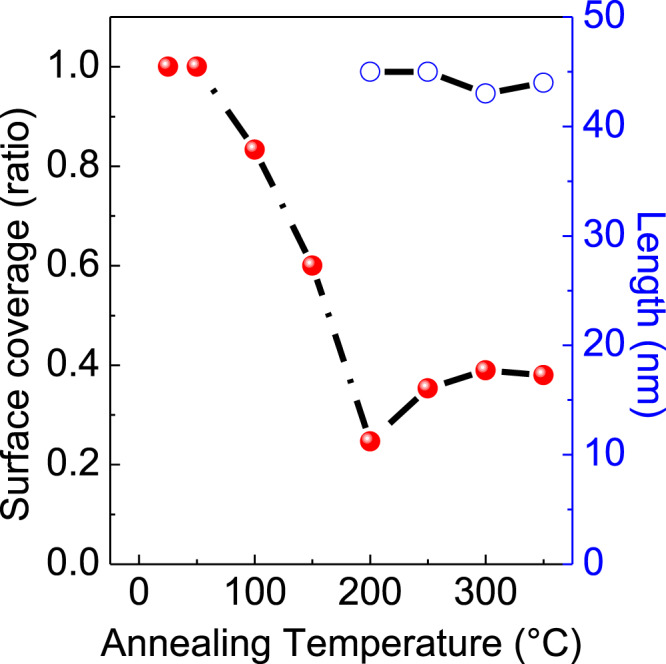
Fractional surface coverage and maximal length of oligomers/7-aGNRs. Annealing temperature dependent fractional molecular surface coverage remained (red dot and line) after each annealing step of 1.5 ML DBBA on the Au(111) surface and maximum oligomer/7-aGNR lengths (blue circles and line) observed between 200 °C and 350 °C annealing steps.

Large-scale STM images at ~4.5 K were recorded on Au(111) surface after the 1.5 ML of DBBA monomers deposition. It clearly shows distinct ML and bilayer (BL, i.e. 2.0 MLs) regions of DBBA monomers (Fig. 2a). In the ML regions, high-resolution STM images reveal unique interdigitated assemblies of DBBA monomers (Fig. 2b). Whereas, on the second layer of BL regions, one-dimensional (1D) linear chain-like assemblies are imaged (Fig. 2c)^28^. The molecular models for the DBBA assembly patterns observed in the 1st and 2nd layers are given in Supplementary Fig. 2. Different samples prepared with 1.5 ML DBBA coverage exhibit the same distinct self-assembly patterns in the 1st and 2nd ML regions. The first annealing step carried out at 50 °C does not reveal any noticeable changes of the assembly patterns in the 1st and 2nd layers at both large-scale and high-resolution STM images. Therefore, we discuss annealing steps starting from 100 °C. The fractional surface coverage extracted at each annealing step from Fig. 3 is plotted as a function of temperature in Fig. 4. This fractional surface coverage governs the specific on-surface chemical reactions occurring for the 1.5 ML initial sample coverage.

### Stepwise annealing experiments for the 1.5 ML DBBA coverage

During the first annealing step of a 1.5 ML on Au(111), at 100 °C, DBBA molecules partially desorb from the BL islands creating herringbone-like patterns on the Au(111) surface (Fig. 3a). The ML regions remain mostly unaffected except for the formation of local point-defect-like protrusions as observed in the STM topographic corrugation (Supplementary Fig. 3). The d*I*/d*V* characteristics recorded near and on top of these protrusions do not reveal any notable tunneling electronic resonances. This indicates that these protrusions are originating from the surface conformational changes of individual DBBA molecules within the self-assembled ML regions of DBBA molecules. A fraction of the BL islands undergoes a much more dramatic molecular reorganization comprising 2D monomer stripe patterns and amorphous aggregates. The resulting partial desorption of the monomers from the BL regions leads to the shrinkage of the BL islands (Fig. 3a) and of the fractional surface coverage (Fig. 4).

For 100 °C ≤ *T* ≤ 200 °C, prior to the polymerization, the decrease in the molecular coverage continues with a complete disappearance of the ML DBBA regions from the surface. At 150 °C, the surface monomer desorption results in a bimodal distribution of smaller BL and larger ML islands (Fig. 3b) and a further decrease of the fractional surface coverage (Fig. 4). Additionally, the surface molecular organization is modified (Supplementary Fig. 4). In ML regions, the density of protrusions increases, while the 1D linear chain-like assemblies of the BL regions are fully converted to 2D periodical stripes and amorphous clumping. These changes reveal a drastic molecular conformational change at the known onset of the polyanthryl polymerization temperature.

**Fig. 5:**
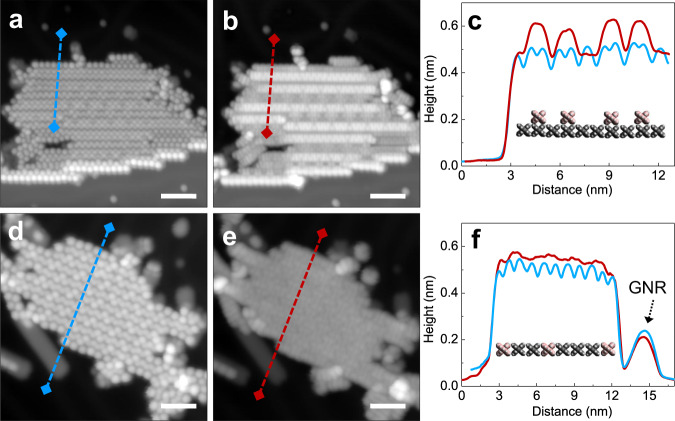
Bias polarity-dependent appearance of polyanthryl oligomer nano-islands on the Au(111) surface. **a**, **b** High-resolution STM topographs recorded after the 200 °C annealing step showing the distinct morphology of an oligomer nano-island at the positive bias of (**a**) + 2.0 V/10 pA and at the negative bias of (**b**) −2.0 V/10 pA. **c** Corresponding line profiles drawn across the oligomer island in (**a**) and (**b**) are presented with a blue and a red curve, respectively. The presence of two STM apparent heights reveals the bilayered configuration of polyanthryl oligomer islands at 200 °C annealing step. A reconstructed molecular model of two-layered oligomer structure is given in the inset. **d**–**f** A similar bias polarity dependent high-resolution STM topograph analysis carried out at the 250 °C annealing step resemble the 200 °C annealing step presented in (**a**–**c**), respectively. The presence of uniform and single STM apparent heights indicates the monolayered configuration of polyanthryl oligomer islands at the 250 °C annealing step. Scale bars are 5 nm in (**a**, **b**, **d**, **e**).

At ~160–200 °C, the Ullmann coupling reaction of DBBA molecules occurs with the formation of single covalent C−C bonds at each 10 and 10ʹ sites along the polyanthryl chains (Supplementary Fig. 5). Figure 3c shows that all the molecular herringbone-like patterns have now vanished, thereby exposing large portions of the bare Au(111) surface, which is complemented by the steep fall in fractional surface coverage from 100% to 24% between 50 °C and 200 °C annealing steps (Fig. 4). At this stage, all molecular chains are gathered into nano-islands with similar lateral sizes (~40–50 nm) and uniform apparent STM heights (Supplementary Fig. 6). The bias polarity dependent high-resolution STM topographic behavior highlighted through Fig. 5a–c reveals the presence of polyanthryl oligomers in the bilayered configuration at the 200 °C annealing step. The observation of bilayered oligomer islands on the Au(111) surface implies that the Ullmann-type coupling occurs simultaneously in both 1st and 2nd layers of precursor monomers. By performing single molecule STM lateral manipulations, individual polyanthryl chains can be extracted one-by-one from the nano-islands suggesting that the DBBA monomers are engaged in the progressive formation of the polyanthryl chains. Further STM molecular manipulations demonstrate that these chains are neither covalently bonded together in a transverse Ullmann-like coupling nor by inter-chain cyclodehydrogenations (Supplementary Fig. 7). After STM manipulations, the STM images are constellated with isolated atom-like defects on the Au(111) surface, certainly coming from the Br atoms released during the Ullmann coupling reaction (Supplementary Fig. 7).

At ~250 °C, the molecular organization observed in the form of oligomer nano-islands with lateral sizes of ~40–50 nm prevails (Fig. 3d). However, high-resolution STM images taken as a function of bias polarity show the absence of the 2nd layer and present a compact side-by-side alignment of oligomer chains with a uniform contrast (Fig. 5d, e). The line profiles across a given nano-island also exhibits similar apparent heights of ~0.51 ± 0.02 nm and ~0.54 ± 0.02 nm at both polarities (Fig. 5f); thereby confirming the single-layered structure of polyanthryl oligomers in the nano-islands. However, reaching 250 °C, an inversion of the fractional surface coverage as a function of the temperature is observed (Fig. 4). The fractional surface coverage is boosted from 24% at 200 °C to 35% at 250 °C. This confirms that the oligomer chains of the 2nd layer diffuse into the 1st layer, increasing the fractional surface coverage (See Supplementary information and Supplementary Fig. 8 for more details about the d*I*/d*V* spectroscopic perception of the BL to ML reorganization of polyanthryl oligomers in a given nano-island). In addition to the single-layered structure of the oligomer islands at 250 °C, the large scale STM image presented in Fig. 3d is also showing isolated molecular chains scattered all over the Au(111) surface. High-resolution STM images (Supplementary Fig. 9) indicate that they are not polyanthryl chains but partially or fully dehydrogenated GNRs with their characteristic planar structure. Figure 3d and Supplementary Fig. 9 also clearly show polyanthryl oligomer chains partially transformed into GNRs and protruding at the periphery of the polyanthryl oligomer nano-islands. This is the result of cyclodehydrogenation in progress along the polyanthryl chains, which is captured here precisely at the adequate surface temperature. In Fig. 5d–f, the STM images also captured an incomplete half-cooked 7-aGNR with a ~0.2 nm apparent height at the periphery of the nano-islands. The completed part of these incomplete GNRs interacts weakly with the polyanthryl nano-islands. Once entirely converted to 7-aGNRs, these molecular wires diffuse away on the reconstructed Au(111) surface. Indeed, the STM imaging also captures such completely transformed and well-isolated GNRs away from the polyanthryl nano-islands, generally trapped at the Au(111) herringbone kink sites and oriented along the reconstruction direction.

**Fig. 6:**
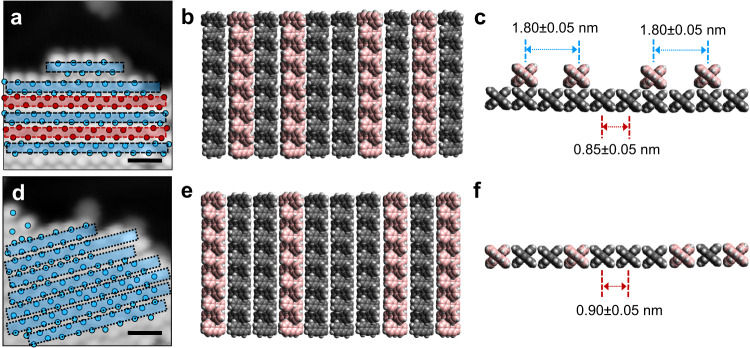
Reconstruction of BL to ML reorganization of polyanthryl oligomers in a nano-island after the 200 °C and 250 °C annealing steps. **a** A scheme overlaid on the high-resolution STM image shows the 1st layer and 2nd layer oligomer chains in a BL configuration of nano-island at 200 °C in blue and red frames, respectively. Each isolated stripe corresponds to an oligomer chain. Molecular model schematics showing (**b**) plane and (**c**) cross-sectional views of two-layered oligomer nano-island correlating the experimental STM topograph configuration in (**a**). **d**–**f** Schematic model representations of reorganized ML configuration of oligomer nano-island at 250 °C in blue frames similar to the ones presented in (**a**–**c**), respectively. Scale bars are 1 nm in (**a**, **d)**.

Prior to polymerization, the DBBA monomer desorption process leads to the preservation of BL islands, in which the 2nd layer slows down the overall monomer desorption rate. Ullmann polymerization takes place in both layers of the resulting BL islands. After polymerization, the oligomer chains undergo reorganization from BL to ML indicating that the cyclodehydrogenation step requires a direct interaction of the oligomer chains with the Au(111) surface. This process is completed between 250 °C and 300 °C. Beyond which, the cyclodehydrogenation of the oligomer chains proceeds at the periphery of an island, where they are in direct contact with Au(111) surface. At the same time, the STM images recorded at 250 °C (Fig. 3d and see Supplementary Fig. 9) show that the peripheral polyanthryl chains of the 1st layer are extruded as cyclodehydrogenated GNRs. These concomitant events account for the disappearance of the 2nd layer of the bilayered nano-islands and for the appearance of isolated 7-aGNRs fully or partially conjugated. This leads to the rebound of the fractional surface coverage reaching later its final plateau of ~40% at 300 °C (Fig. 4). In Fig. 6, the structural models represent the transformed two-layered to one-layered oligomer nano-island between 200 °C and 250 °C. The lateral chain periodicity is ~0.90 ± 0.05 nm at 250 °C, in good agreement with the value measured at the previous 200 °C annealing step.

When reaching 300 °C, all nano-islands have been converted into isolated 7-aGNRs as presented in Fig. 3e. Stopping the on-surface chemical reaction at 300 °C reveals that the cyclodehedrogenation of shorter GNRs (length up to 5–8 nm) is completed. Longer GNRs retain hydrogenated segments easily identified by their bright contrast at one side of the molecular structure (Supplementary Fig. 10). Here, some of the anthracene units along the polyanthryl chains are still hydrogenated^22^.

**Fig. 7:**
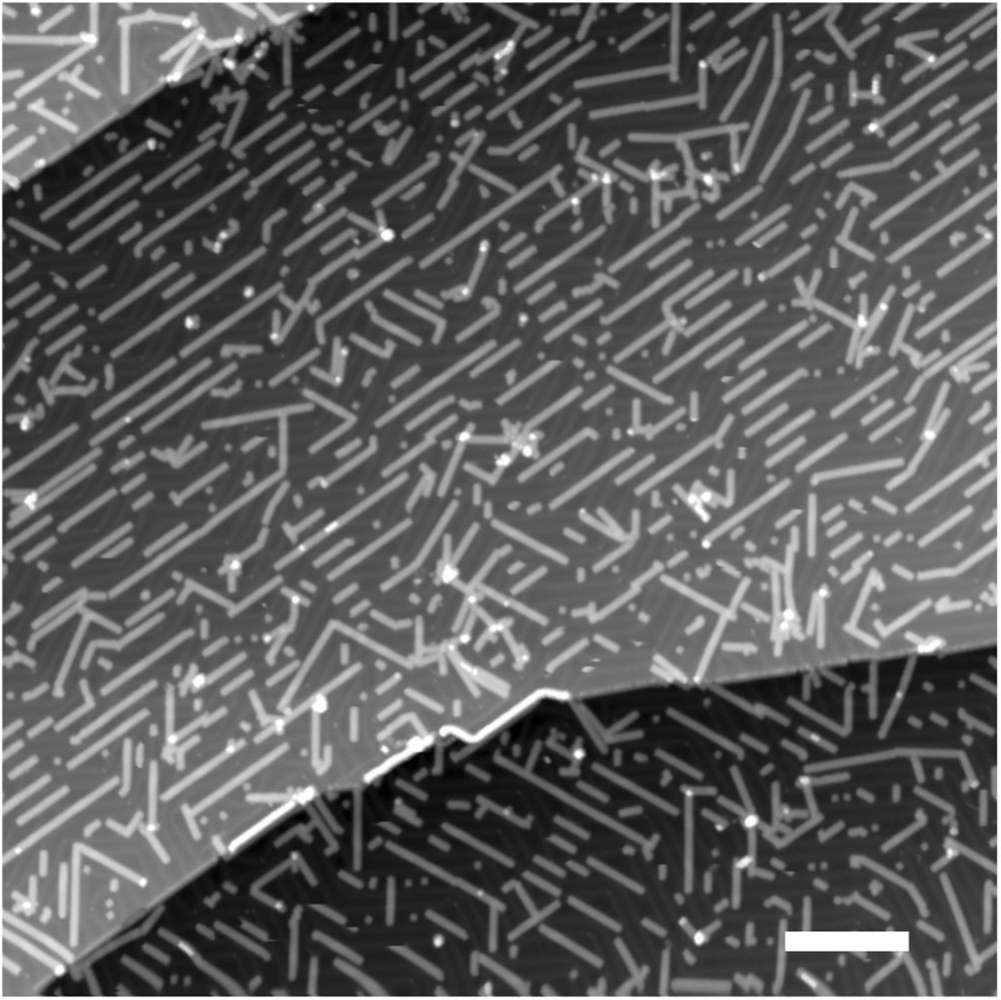
Large-scale STM image recorded after the final step of on-surface synthesis protocol. Large-scale STM image after the 350 °C annealing step showing completely transformed, aligned and well-isolated 7-aGNRs on the Au(111) surface. STM set parameters are −2.0 V/10 pA. Scale bar is 25 nm.

At 350 °C, the final annealing of the same sample used during this entire step-by-step temperature follow-up completes the cyclodehydrogenation process for all 7-aGNRs, independently of their length (Figs. 3f, 7). Higher resolution STM image, d*I*/d*V* spectroscopy and maps, performed on a given isolated 7-aGNR derived from 5 DBBA monomer units (Supplementary Fig. 11), confirm the presence of the edge states (R0) near the Fermi level (zero bias in STM). The first positive (R + 1) and negative (R − 1) tunneling resonances are the signature of a perfect and atomically precise 7-aGNR^9,29,30^.

## Discussion

Starting with 1.5 ML coverage and after each annealing step, the fractional surface coverage controls the on-surface chemical reaction kinetics (red dots and line in Fig. 4). This control can only be reached when the well-known two-step on-surface GNR synthesis is sequenced with a room temperature quench after each annealing temperature step, while the sample remains in UHV. This step-by-step annealing protocol and room temperature re-thermalization lead to the on-surface synthesis of long and well-isolated individual 7-aGNRs. It has to be compared with the commonly reported dense coverage (more than a monolayer) of 7-aGNRs based on the standard two-step approach with no intermediate room temperature quench^22–24^. With this on-surface chemical synthesis protocol, it appears that stopping the on-surface chemical reaction at each 50 °C and returning to room temperature at each step is cleaning the Au(111) surface from the light residues of the previous steps beneficiating from the UHV. Freezing the on-surface chemical reaction at each 50 °C also offers an advantage to stop any possible side-way reaction and/or surface diffusion.

The length distributions of the oligomer chains at 200 °C and of the 7-aGNRs at 350 °C are reaching the same maximal length of ~40–50 nm (Fig. 4, blue circles and line). The average lateral size of an oligomer island at 200 °C is also around ~50 nm, which is commensurate with the observed maximum length of the oligomers and of the final 7-aGNRs. Our initial DBBA monomer bilayer islands configuration is acting like a surface molecular mold developed by the desorbed ML regions during the annealing processes. The DBBA BL assembly islands are assisting the polyanthryl oligomer formation and are also pre-setting the 7-aGNR length distribution. With an initial DBBA coverage exceeding 2.0 MLs, following the same annealing protocol results in very densely packed GNRs (>1.0 ML) with no bare Au(111) surface.

As alternative attempts to GNR lengthening, we have considered and tested several experimental variations (Supplementary Fig. 12) around our protocol such as longer-time annealing (60 min) at temperatures slightly below (at ~180 °C) the polymerization temperature of DBBA monomers (protocol A), depositing the DBBA directly at the polymerization step (protocol B), reducing the DBBA deposition rate as slow as 0.01 ML/min (~200 °C surface temperature, protocol C) and increasing it as high as 0.33 ML/min (180 °C crucible temperature, protocol D). Additionally, we have fed the surface with extra monomers by re-depositing more DBBA (about 1.0 ML of room temperature surface deposition) at the polymerization step with the substrate at ~200 °C (at polymerization temperature, protocol E). None of these conditions resulted in any significant improvement in the maximal lengths of oligomer chains or GNRs. From this wide range of conditions, it appears that an initial monomer surface coverage close to 1.5 ML suffices to maximize the GNR length and is self-limiting for DBBA.

Our detailed on-surface synthesis experiments point out the need for new monomers^1,4,31^ and protocols^14,15,32,33^ on different surfaces^34–37^ dedicated to monomolecular electronics that potentially increase the size and optimize the shape of BL islands at the polymerization temperature to produce a low coverage of well-isolated and even longer GNRs.

## Methods

### Sample preparation

Au(111) single crystals (*Mateck* GmbH) were used for the on-surface synthesis of 7-aGNRs. All samples were prepared in the preparation chamber of our LT-UHV 4-STM instrument. Au(111) surfaces were cleaned under UHV conditions by repeated cycles of Ar^+^ ion sputtering at 1.0 keV and ~10^−6^ mbar for about 10 min and subsequent annealing at about 740 K for 1 h. The cleanliness, the herringbone reconstruction and the presence of the characteristic surface state of the Au(111) were checked using one of the STM of our LT-UHV 4-STM operated at sample temperatures of ~4.5 K. Precursor monomers, DBBA from *Aldrich*, were thermally evaporated from the quartz crucible of a *Kentax* thermal evaporator (*Knudsen*-cell) at 160 °C while the clean Au(111) surface was kept below 40 °C and ca. 50 mm away from the evaporation source. Prior to the deposition, the monomers were degassed for more than 24 h in UHV to remove any moisture and oxygen contaminants present in the crucible. The above deposition parameters yielded 0.5 ML, 1.0 ML, and 1.5 ML of DBBA in 5, 10, and 30 min, respectively (effective coverage measured using large-scale STM images). We observe that the effective growth rate deviates from a linear time dependence after exceeding 1.0 ML of DBBA coverage, which might be due to the difference between a monomer/Au(111) (<1.0 ML DBBA) and monomer/monomer/Au(111) (>1.0 ML DBBA) surface adsorption under UHV conditions.

### STM and STS measurements

All samples were transferred under UHV conditions and characterized in situ with the LT-UHV 4-STM instrument (base pressure is <3 × 10^−11^ mbar). Electrochemically etched PtIr (Ir: 10%) tips were used for STM and STS characterization. All bias voltages mentioned are with respect to the tip, with the sample virtually grounded.

### Supplementary information

Supplementary Information

## Data Availability

The data reported by this article are available from the corresponding author upon reasonable request.
