# Supplementary material for: Long and isolated graphene nanoribbons by on-surface polymerization on Au(111)
Source: Commun Chem. 2023 Dec 6;6:266. doi: 10.1038/s42004-023-01073-3 (PMC10700363; doi:10.1038/s42004-023-01073-3)
Supplement: Supplementary file 1 — Supplementary Information [file 42004_2023_1073_MOESM1_ESM.pdf]

SUPPLEMENTARY INFORMATION

Long and isolated graphene nanoribbons by on-surface polymerization on Au(111)

Umamahesh Thupakula,[1,] We-Hyo Soe,[1,†] Christian Joachim,[1,2] and Erik Dujardin,[1,3]*

[1]Centre d'Élaboration de Matériaux et d'Études Structurales (CEMES), Centre National de la Recherche Scientifique (CNRS), Université de Toulouse, 29 Rue J. Marvig, BP 94347, 31055 Toulouse Cedex, France.

[2]International Center for Materials Nanoarchitectonics (WPI-MANA), National Institute for Materials Science (NIMS), 1-1 Namiki, Tsukuba, Ibaraki 305-0044, Japan.

[3]Laboratoire Interdisciplinaire Carnot de Bourgogne, CNRS UMR 6303, Université de Bourgogne Franche-Comté, 9 Av. A. Savary, 21078 Dijon, France.

[†]Present Address: Center for Quantum Nanoscience, Institute for Basic Science (IBS), Seoul 03760, Korea

Ewha Womans University, Seoul 03760, Korea

* Corresponding author Email: umamahesh.thupakula@cemes.fr

umamahesh.thupakula@gmail.com

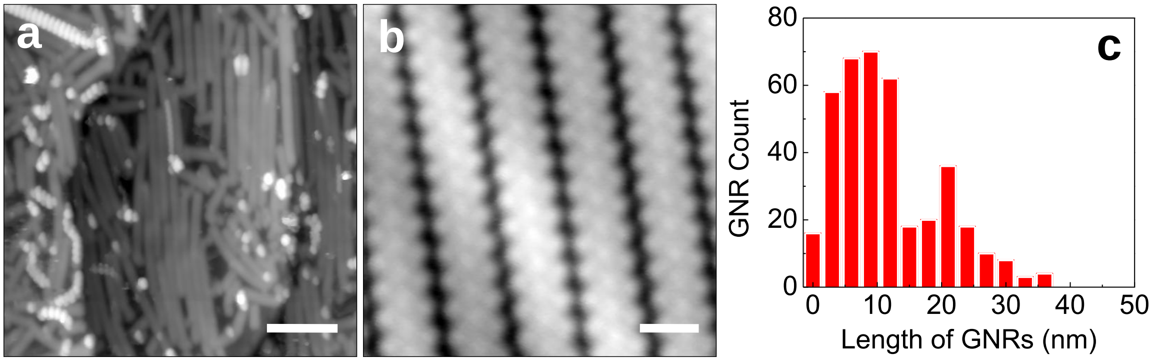

Supplementary Figure S1. Effect of very large initial DBBA coverage on the final GNR lengths. (a) Large-scale and **(b)** high-resolution STM topographs showing the close packed assembly of GNRs obtained after annealing the sample with more than 2 monolayers (MLs) of DBBA monomers initially deposited on the Au(111) surface. **(c)** GNR length histogram revealing the maximum length of GNRs is ~40 nm. STM set-parameters are –0.4 V/100 pA. Scale bars are 10 nm for **(a)** and 1 nm for **(b)**.

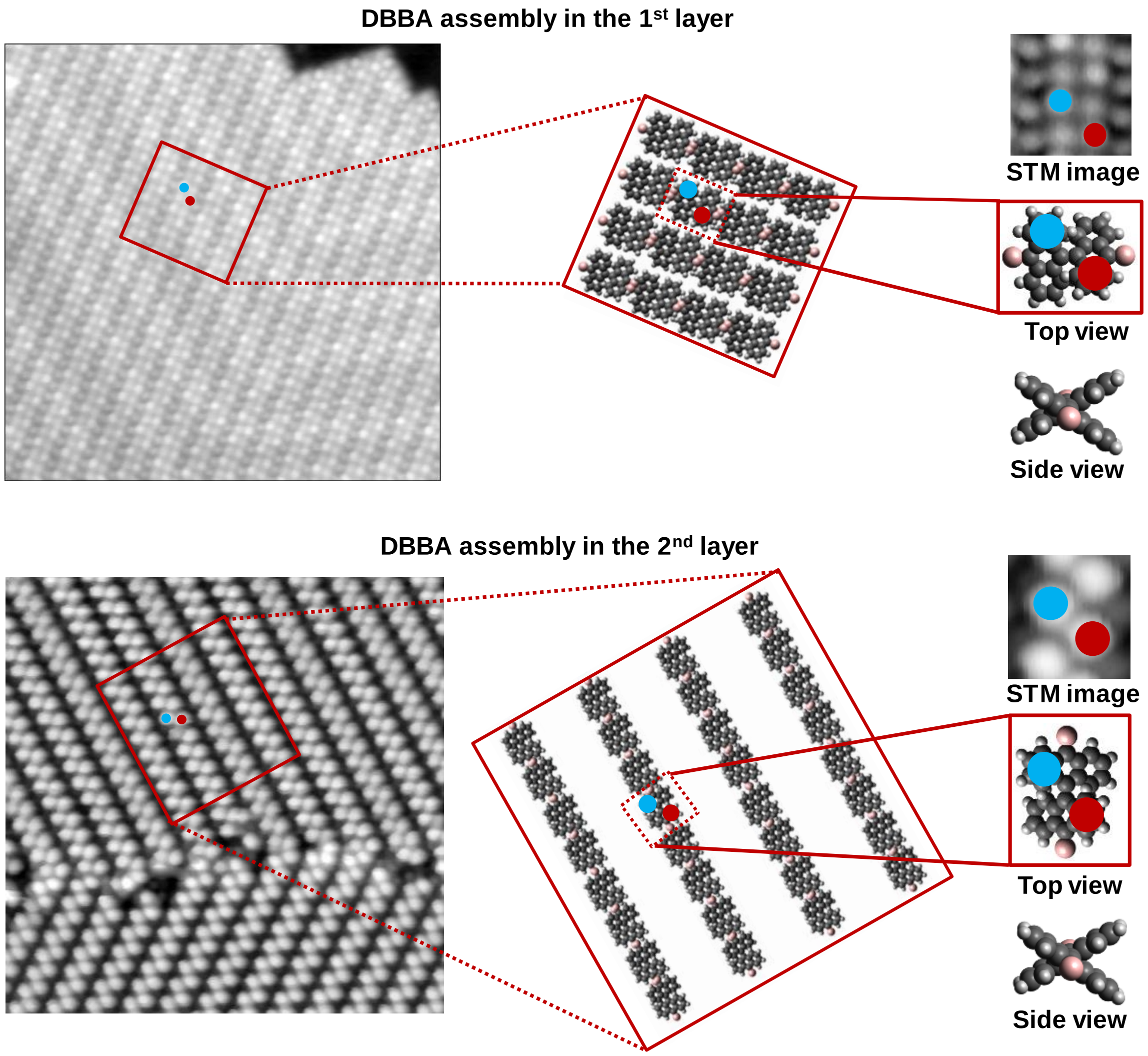

Supplementary Figure S2. Molecular model interpretation of the DBBA assembly on Au(111) surface. Top panel: Starting from the original LT-UHV STM image (left, from Fig. 2b of main text), the schematic molecular model reconstructing the interdigitated DBBA self-assembly in the 1st layer (middle) and single molecule representations (right). Bottom Panel: Similar molecular model reconstructions of the linear chain like self-assembly of DBBA in the 2nd layer on Au(111) surface, comparing the STM image (from Fig. 2c of main text) with single molecule model representations. Large scale STM images (left): 25 nm × 25 nm (scan parameters are +2 V/10 pA). High-resolution STM images (top right): 2 nm × 2 nm (scan parameters are +2 V/10 pA) The high conductance spots (blue and red dots) correspond to the up tilted phenyls of DBBA. In the DBBA assembly of 2nd layer, only the 2nd layer side view is indicated.

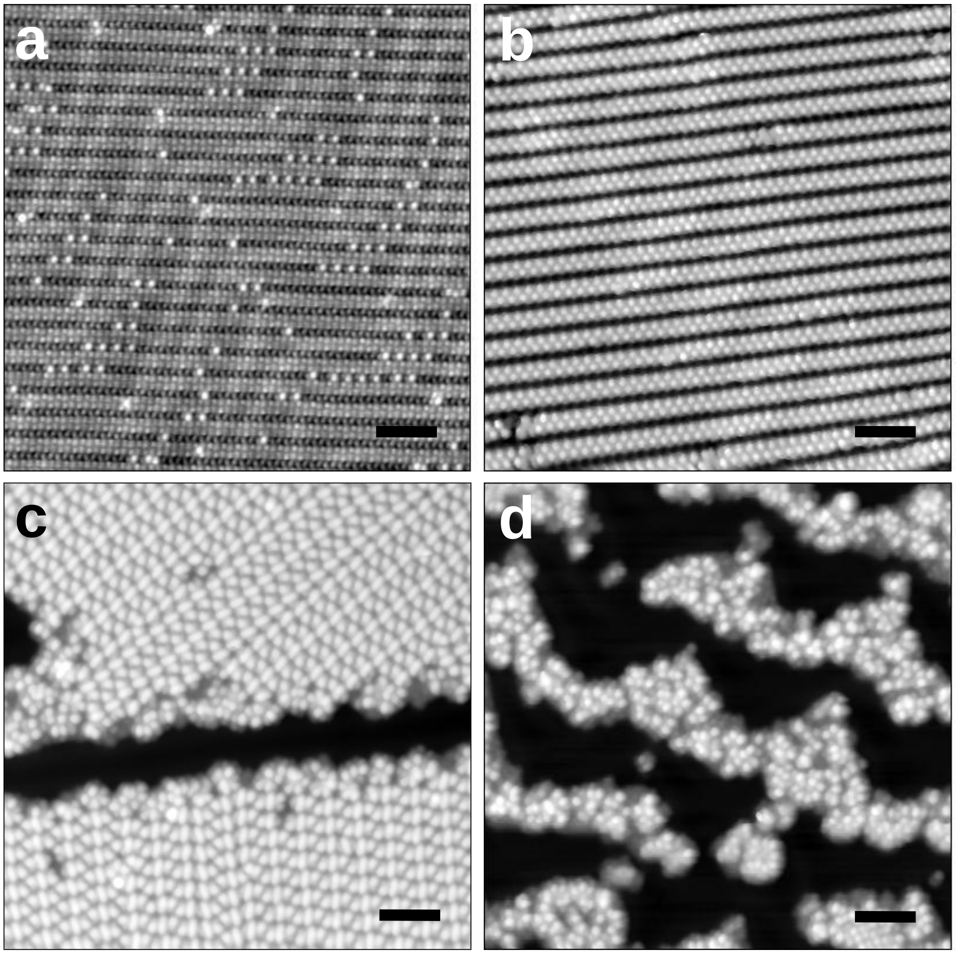

Supplementary Figure S3. Surface morphology after the 100 °C annealing step. STM topographs revealing DBBA assembly patterns in **(a)** ML and **(b-d)** bilayer (BL) regions, after annealing the 1.5 ML DBBA/Au(111) sample at 100 °C for 30 minutes. ML regions show atomic-defect-like protrusions, which originate from the physical conformational changes of individual DBBA molecules within the assembly. BL regions presented in **(c)** and **(d)** show partial reorganization of DBBA assembly differing from the initial 1D chain like structure shown in **(b)**. STM set-parameters are +2 V/10 pA. Scale bars are 5 nm.

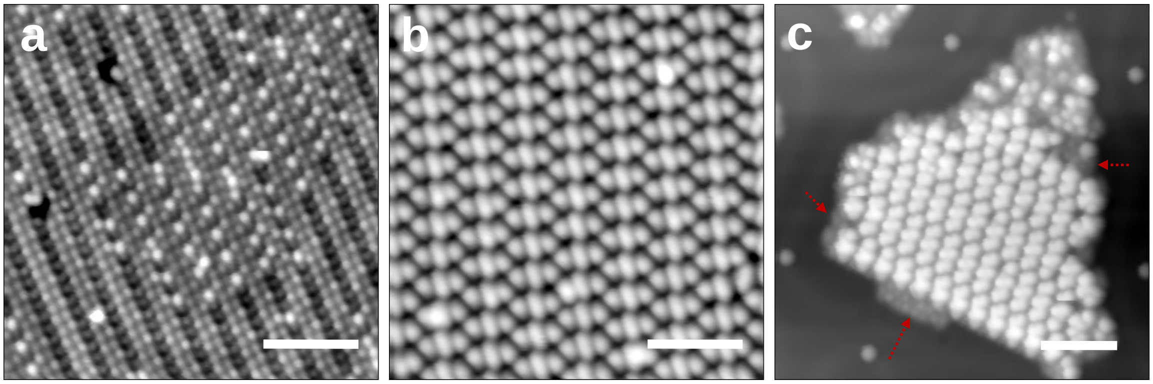

Supplementary Figure S4. Surface morphology after the 150 °C annealing step. (a) STM topograph showcasing the point-defect-like protrusions in the ML regions of DBBA on the Au(111) surface. **(b)** STM topograph showing a BL region revealing the fully reorganized 2nd layer of DBBA on the Au(111) surface. **(c)** STM topograph of a BL DBBA island showing the intact 1st layer (red arrows) below the reorganized 2nd layer. STM set-parameters are +1 V/10 pA for **(a, c)** and +2 V/10 pA for **(b)**. Scale bars are 5 nm.

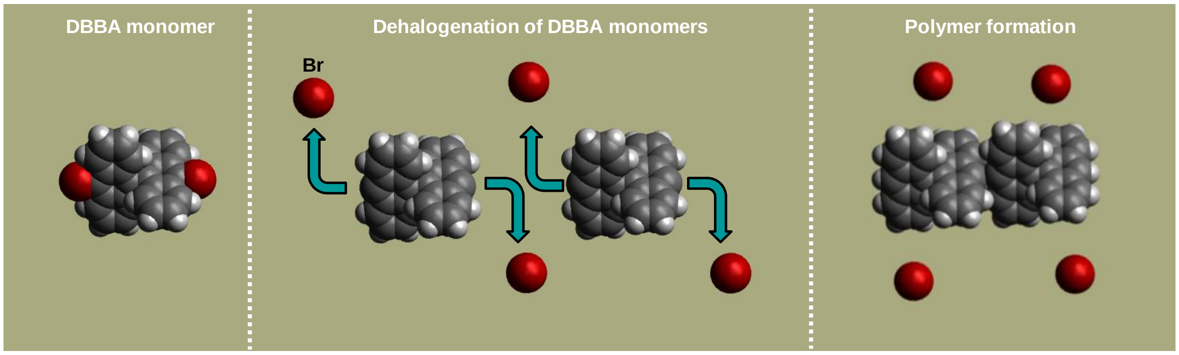

Supplementary Figure S5. Ullmann type coupling chemical reaction of DBBA monomers. Molecular model schematics showing the individual DBBA monomer unit (left), dehalogenation of monomers (middle) and polyanthryl oligomer formation (right). Carbon, bromine and hydrogen are indicated with gray, red and white balls, respectively.

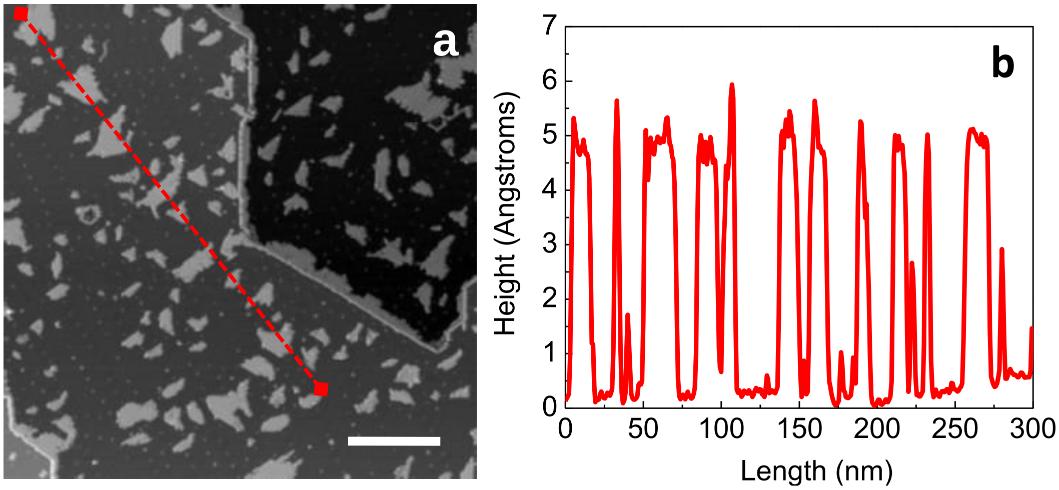

Supplementary Figure S6. Surface morphology after the 200 °C annealing step. (a) Large-scale STM topograph and **(b)** height profile along the dashed line in **(a)** showing the uniform thickness of oligomer nano-islands on the Au(111) surface. STM set-parameters are +2 V/10 pA. Scale bar is 60 nm.

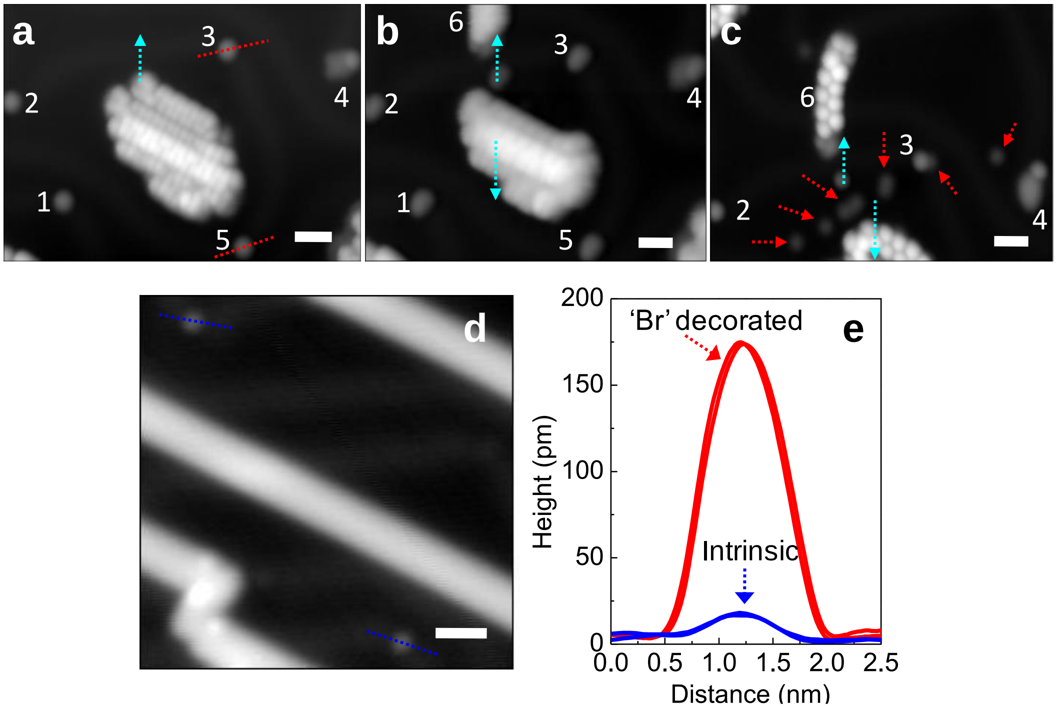

Supplementary Figure S7. (a-c) STM topographs showing the manipulation sequence and underlying atomic features below the manipulated polymer island. Markers 1 to 6 are indicated in the STM images **(a-c)** to facilitate the visualization of the molecule displacements. Cyan arrows indicate the polymer island manipulation direction. Red arrows indicate the atomic features appearing after the manipulation process. We attribute these atomic features to the bromine released from the monomers at the onset of Ullmann coupling reaction. **(d)** STM topograph showing the typical herringbone Au(111) kink defects after the 350 °C annealing step. **(e)** Height profiles across the kink defects after the 200 °C (red corresponding to the dotted lines in **(a)**) and 350 °C (blue corresponding to the dotted lines in **(d)**) annealing steps. The apparent enlargement of defect sizes after the 200 °C annealing step (red) is clearly identified and can be attributed to the bromine decoration. STM set-parameters are –2 V/10 pA for **(a, b and d)** and +2 V/10 pA for **(c)**. Scale bars are 2 nm.

d*I*/d*V* spectroscopic perception of the BL to ML reorganization of polyanthryl oligomers. A lock-in detection technique was implemented to carry out the STS measurements by recording both the d*I*/d*V* spectra as well as the d*I*/d*V* maps with open feedback loop conditions (V_{mod} = 16 mV or 20 mV at ~430 Hz).

Detailed d*I*/d*V* spectra recorded on the bilayered polyanthryl oligomer nano-islands just after reaching 200 °C annealing step give the origin of different tunneling channels through the 1st or the 2nd layer of the polyanthryl oligomers. Below −1.0 V, the d*I*/d*V* spectra recorded on top of a 1st and 2nd oligomer layer exhibit distinct tunneling electronic resonances (Fig. S8a). The spectrum on top of 2nd layer comprises multiple sharp peaks coming from the inelastic excitation of vibrations in the oligomer chain.[1] The spacing between the vibronic replicas (labeled V_1, V_2, V_3 and V_4) indicates the dominant vibrational mode with an energy of ~200 meV. Such vibronic satellite peaks are absent in the spectrum recorded on top of the 1st layer oligomer chain and thereby presenting only one broad resonance around ~ −1.4 V. Consistently, the d*I*/d*V* map recorded at ~ −1.1 V (i.e. at the V_1 resonance) shows an enhanced contrast coming from the 2nd layer oligomers (Fig. S8b). The other d*I*/d*V* maps recorded at the bias voltages of the satellite peaks V_2 and V_3 have an identical appearance, thus confirming the vibronic origin of these peaks.[2] On the other hand, the d*I*/d*V* spectra and maps recorded on a given polyanthyl oligomer nano-island at 250 °C annealing step also confirm the disappearance of the vibronic satellite peaks that were originating from the 2nd layer of oligomer chains (Figs. S8c and S8d). This further confirms the BL to ML reorganization of oligomer nano-island between 200 ° C and 250 ° C annealing temperatures.

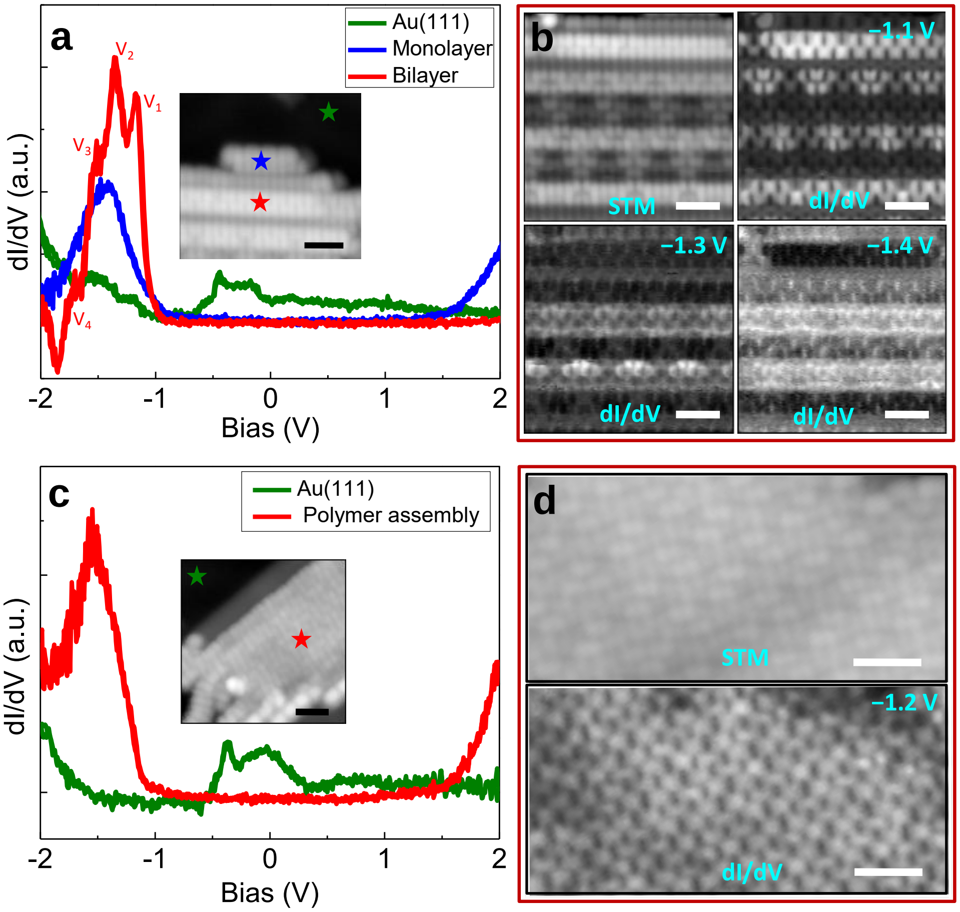

Supplementary Figure S8. Scanning tunneling spectroscopy of a polyanthyl oligomer island. (a) d*I*/d*V* spectra recorded on top of the 1st layer (blue line) and the 2nd layer (red line) oligomer chains of an island observed after the 200 °C annealing step. The reference d*I*/d*V* spectrum recorded on the bare Au(111) surface region is presented with a dark green line. Corresponding STS positions are marked with respective colored stars in the STM topograph presented in the inset. The vibronic satellite tunneling resonances (V_1, V_2, V_3, and V_4) from the 2nd layer oligomer chain are labeled in the d*I*/d*V* plot. **(b)** d*I*/d*V* maps recorded at three different voltages (−1.1 V, −1.3 V and −1.4 V) suggest the vibronic origin of the satellite peaks. **(c)** d*I*/d*V* spectrum (red) and **(d)** map recorded from a polyanthyl oligomer island after the 250 °C annealing step. The reference d*I*/d*V* spectrum in green is identical to the green plot in **(a)**. No vibronic satellite peaks from oligomer chains are observed but the spectrum is identical to the blue plot in **(a)**, implying that the oligomer island has converted to a single layered configuration. STM set-parameters are –2 V/10 pA for the STM images given in the insets of **(a)** and **(c)**, –1.1 V/100 pA for **(b)** and –1.2 V/100 pA for **(d)**. Scale bars are 2 nm.

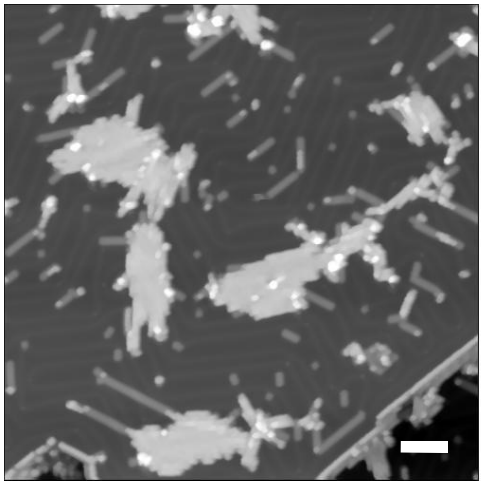

Supplementary Figure S9. Surface morphology after the 250 °C annealing step. STM topograph showing the cyclodehydrogenation process occurring for the oligomer chains located at the periphery of the island. The transformed part of the GNR can be found away from the island. STM scanning set-parameters are –2 V/10 pA. Scale bar is 10 nm.

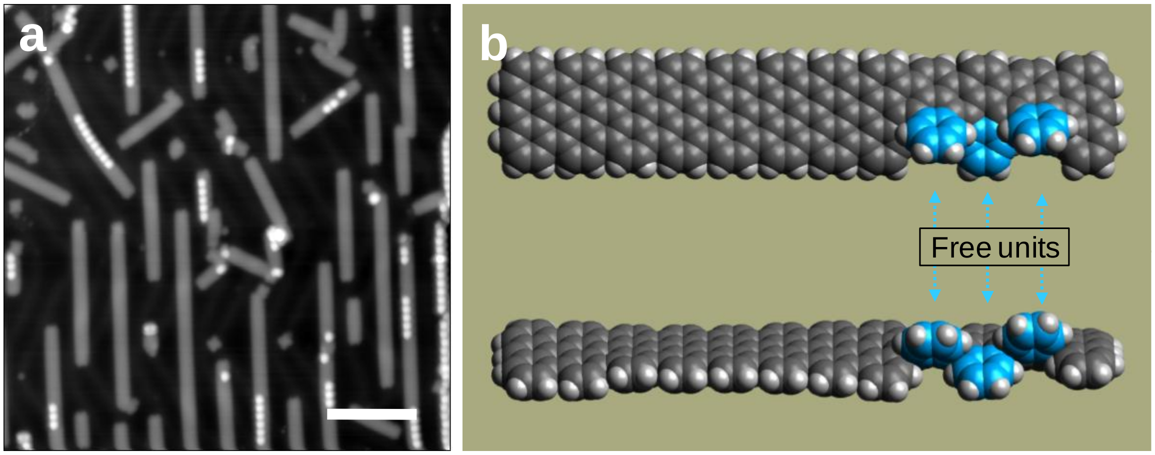

Supplementary Figure S10. Surface morphology after the 300 °C annealing step. (a) STM topograph showcasing the partially transformed GNRs with un-reacted benzene rings on one side of the ribbon. STM set-parameters are –2 V /10 pA. Scale bar is 5 nm. **(b)** Molecular model schematic showing the planar (top) and the side (bottom) views of a partially transformed GNR. Un-reacted benzene rings are indicated in blue color.

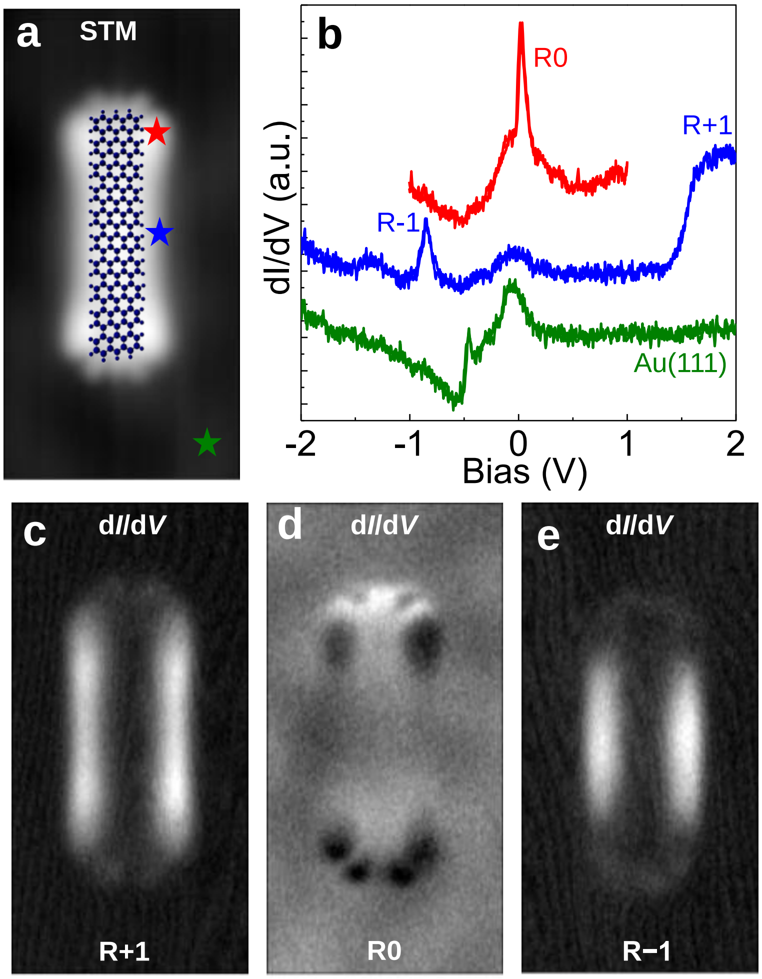

Supplementary Figure S11. d*I*/d*V* characteristics of individual GNR. (a) An STM image of a 5-monomer unit long 7-aGNR with an overlaid molecular model schematic. **(b)** The characteristic d*I*/d*V* tunneling spectra of 7-aGNR recorded at the spectroscopic positions marked with colored stars in **(a)**. A characteristic Au(111) reference spectrum is also shown in green. Corresponding d*I*/d*V* maps revealing the characteristic first resonances of 7-aGNR at **(c)** the positive (R+1) and **(e)** the negative (R−1) bias voltages and, **(d)** the edge state resonance (R0).

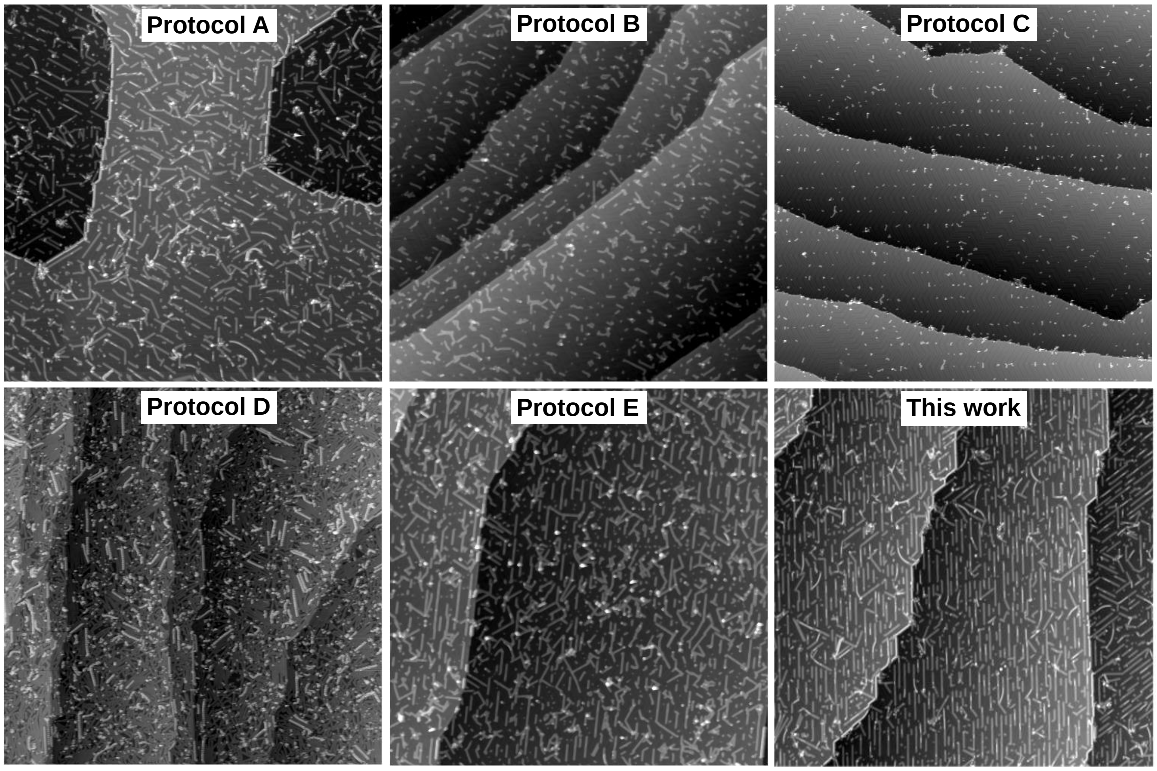

Supplementary Figure S12. Large-scale STM images showing the GNR length dispersions obtained through different on-surface protocols described in the main text, comparing with the present work (lower right STM image). Scan areas are: 300 nm × 300 nm. Scan parameters are: −2 V/10 pA.

References

1. Schulz, F. et al. Many-body transitions in a single molecule visualized by scanning tunnelling microscopy. Nat. Phys. 11, 229−234 (2015).

2. Lit, J. et al. Suppression of electron-vibron coupling in graphene nanoribbons contacted via a single atom. *Nat. Commun.* **4**, 2023 (2013).
